# Comparative host selection responses of specialist (*Helicoverpa assulta*) and generalist (*Helicoverpa armigera*) moths in complex plant environments

**DOI:** 10.1371/journal.pone.0171948

**Published:** 2017-02-09

**Authors:** Wei-zheng Li, Xiao-hui Teng, Hong-fei Zhang, Ting Liu, Qiong Wang, Guohui Yuan, Xian-ru Guo

**Affiliations:** Department of Entomology, College of Plant Protection, Henan Agricultural University, Zhengzhou, Henan Province, China; Rutgers The State University of New Jersey, UNITED STATES

## Abstract

We tested the behavioral responses of ovipositing females and natal larvae of two sibling species, a generalist *Helicoverpa armigera* (Hübner) and a specialist *Helicoverpa assulta* (Guenée), to odor sources emitted from different combinations of six plant species (tobacco, *Nicotiana tabacum*; hot pepper, *Capsicum annuum*; tomato, *Solanum esculentum*; cotton, *Gossypium hirsutum*; peanut, *Arachis hypogaea*; maize, *Zea mays*). Under the conditions of plant materials *versus* corresponding controls, both stages of both species could find their corresponding host plants. However, *H*. *assulta* females and larvae exhibited a supersensitive and an insensitive response, respectively. Under the conditions of tobacco paired with each plant species, *H*. *assulta* females exhibited more specialized ovipositional response to tobacco than its sibling. When each plant species were combined with tobacco and tested against tobacco reference, peanut played an opposite role in the two species in their ovipositional responses to tobacco, and cotton can enhance the approaching response of *H*. *armigera* larvae when combined with tobacco. It seems that two attractive host plants also can act antagonistically with respect to host selection of the generalist via volatile exchange. Tomato should better be excluded from host list of *H*. *assulta*.

## Introduction

Intercropping system design based on natural ecosystem mimicry has been becoming a hot-point of sustainable pest management, which calls for in-depth knowledge about host selection process of phytophagous insects under a highly complex odorant background [1–2]. Two major approaches, field observation and laboratory bioassay, have often been used in the studies intended to interpret why fewer pests are found on host plants growing in more diverse backgrounds than on those in monoculture. A number of confounding factors would inevitably be introduced in field observations [3]. While many laboratory bioassays using various solvent extracts or isolated compounds from plant parts as odor sources, rather than living plants, provided poor insights into natural host selection by ovipositing females of phytophagous insects. Generally, host selection of adult females follows a sequence of searching, orientation, encounter, landing, leaf surface evaluation, and host acceptance [4]. As recently reported, one or more stages mentioned above would be affected via volatile exchange between a host plant and its neighbors, irrespective of their health statuses ([5], and References therein).

Despite agricultural economic importance of the genus *Helicoverpa*, its host selection process in a complex plant environment has received little attention. *Helicoverpa armigera* (Hübner) and *Helicoverpa assulta* (Guenée) are two siblings in this genus. The former is a highly polyphagous species [6], while the latter is specialized on several solanaceous species, mainly tobacco and hot pepper [7]. Although active plant volatiles for attracting or stimulating oviposition of *H*. *armigera* [8–10] and *H*. *assulta* [7, 11] has been reported from an agricultural point of view, only a handful of studies seem helpful in understanding the mechanism of their host selection behaviors in a complex odor background.

In the present study, we investigated the female ovipositional preferences and the neonatal approaching responses of these two species to the odor sources from different combinations of six plant species (see below). We will address the questions regarding the discrepancies of the host selection responses between the two species and between the two stages (adult females and their offsprings) within each species, in different plant environments. Specifically, we tested three hypotheses as follows: (1) all the test insects, irrespective of their stages or species, can precisely select their corresponding host plants when test plant materials are paired with neutral substrates; (2) the specialist will exhibit more “specialized” host selection behavior than the generalist, when the shared host (tobacco) was paired with each of the other plants. That is, the former prefer tobacco to the other plants, while the latter not; and (3) the volatiles emitted from the other plants neighboring to tobacco will significantly alter the host selection behavior of test insects to tobacco. Elucidation of the effect of volatile chemical interaction between neighboring plants on host selection of herbivorous insect species may contribute to the optimization of crop spatial arrangement.

## Materials and methods

### Insects

Larvae of *H*. *armigera* and *H*. *assulta* were collected from tobacco and hot pepper fields, respectively, in Scientific & Educational Campus of Henan Agricultural University, Zhengzhou, China. In the laboratory, larvae were fed with corresponding fresh host foliages as originated in the field until pupation, and pupae were sexed according to pupal morphology. Emerged moths were collected daily in cages covered with gauze as an oviposition substrate, and provided with 10% sucrose solution. Newly hatched larvae of subsequent generations were reared in groups on a wheat germ-based artificial diet [12]. After developed into the third instar, the larvae were separated in individual glass tubes (2.0 cm ID×8.0 cm) to prevent cannibalism. The condition of the rearing chamber was set as follows: the temperatures in the light (15 hours) and dark periods (9 hours) were 30 ± 2°C and 26 ± 2°C, respectively, with lights off at 23:00 o’clock; the relative humidity was set at 70 ± 10%.

### Plants

Six plant species were used: Tobacco (*Ncotiana tabacum*; var. NC89), hot pepper (*Capsicum annum*; var. Zhongshu 6), tomato (*Solanum esculentum*; var. Jinpeng 8), cotton (*Gossypium hirsutum*; var. Yumian 19), maize (*Zea mays*; var. Yuyu 30), and peanut (*Arachis hypogaea*; var. Luhua 9). Naturally, all of these crops have been recorded as host plants of *H*. *armigera*, while only tobacco and hot pepper have been confirmed as host plants of *H*. *assulta*. Although several plants in the genus *Lycopersicon*, *Physalis*, and *Solanum* also have been reported as host plants of *H*. *assulta* ([12–13], and References therein), more detailed studies are required to clarify the host status of tomato [14]. All the plants were routinely grown in 2L plastic pots in a greenhouse, in the Scientific & Educational Campus of Henan Agricultural University, Zhengzhou, China. To avoid confusion, the plants will be referred to by their common names hereinafter.

### Choice response of ovipositing females

**Fig 1:**
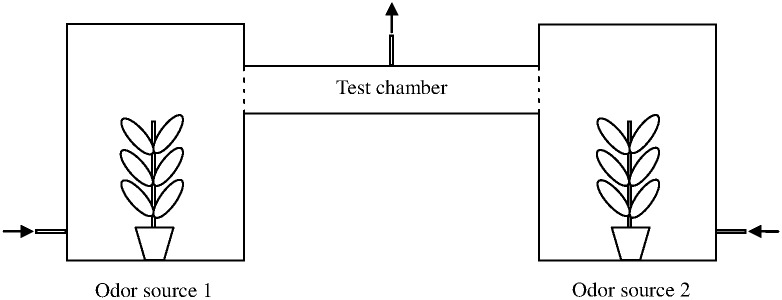
The apparatus for testing the dual-choice responses of ovipositing females. Potted plants or their combinations were placed in the two vertical cylinders, and test moths were released into the test chamber. Dotted lines between the test chamber and the two vertical cylinders indicate two pieces of cotton gauze covered at both ends of the test chamber, which were used as ovipositional substrate. Arrows indicate directions of air movement.

Apparatus for testing the ovipositional choice response of adult females of the two *Helicoverpa* species was modified from Ramasamy et al. [10]. It was constructed of a horizontal Perspex cylinder (Length: 1.0 m; Inner diameter: 15 cm; Outer diameter: 16 cm), which was used as test chamber, and two vertical Perspex cylinders (80 cm height×60 cm ID), which were used for offering plant odor sources. To exclude possible confounding effects of visual and tactile cues, we covered two pieces of appropriate-sized cotton gauze on the both open ends of the horizontal cylinder, which also was used as an oviposition substrate. To avoid possible alteration of plant volatile profile via root interaction, test plants were planted in separate aluminum foil-wrapped pots, and applied additively when complex odor source was needed. Six hundred mL/min air was suck out continuously from a 1 cm diameter hole drilled in the centre of the horizontal cylinder, giving the moths’ equal opportunity to fly to either direction. Two holes were drilled on the opposite sides of each vertical cylinder. The one was 16 cm in diameter, 50 cm height from the base, fitted compatibly with the horizontal cylinder; the other was 1 cm in diameter, 10 cm height from the base, as an inlet of charcoal-filtered, clean air. In each test, the airflows of the two air inlets were carefully regulated to equal with two flow-meters, depending on different air resistance in the two vertical cylinders (Fig 1).

Bioassay was conducted at 18: 00 in an air-conditioned room with ambient temperature 25°C. Undamaged plants, together with their pots, were placed at the bases of the vertical cylinders. Tested plants were non-flowering, all at a height of about 30 cm but peanut. Additionally, it is impossible to test two intact plants of different species with the same shape and leaf area, but care was always taken to minimize such differences through planting time and selecting plants of similar size. Peanut plants were raised to the same height as the other five plants. Three female moths aged three- to four-day and without experience of any plant materials or mates, together with three conspecific males of the same age, were released in the test chamber, and then distilled-water-moistened cotton gauze covered. These ages was used because that mating and egg-laying peaked at these ages, thus sufficient eggs for statistical analysis could be collected in a single scotophase. The entire set-up was covered with black cheese-cloth and air circulation started. Eggs deposited on the cotton gauze were counted at 8: 00 a.m. in the next morning, and a few eggs deposited on the sidewall of the test chamber were excluded from statistical analysis. After each run, the apparatus was dismantled, and its component parts were thoroughly cleaned using absolute alcohol, followed by rinsing in distilled water, to remove any residual odors. The positions of odor sources were alternated between two consecutive runs within a replicate.

### Approaching choice response of newly hatched larvae

A piece of moist filter paper with appropriate size was placed on the bottom of a 14.0 cm ID Petri dish. Based on the experimental design as follows, different combinations of 1.5 cm ID leaf discs or similar-sized green filter paper discs were arranged as option 1 and option 2 along a diameter of the Petri dish. One newly hatched larva (<24 h) was carefully released in the center of the Petri dish, and the lid was covered. Illumination was provided by an overhead fluorescent lamp (10 W, Philips Master LEDtube G13) suspended 50 cm from the Petri dish. We recorded the chose option once the larva firstly contacted with either option. A useful adjunct would have been to score additional behaviors such as staying, feeding, and leaving etc. However, to bring larval bioassay herein into correspondence with that of adult females, we focus on the choice response of naïve larva to volatile cue alone, so feeding bioassay (which is often involved in gustatory and tactile cues) and observation of subsequent behaviors are beyond the scope of this article. After each five runs, the positions of the two options were alternated, and leaf discs were renewed.

### Experimental design

In the whole experiment, *H*. *armigera* and *H*. *assulta* were tested separately, and each species included bioassays of both stages (adult females and the neonates). Within each stage, three sets of bioassays were conducted as follows, and all the replications in each test are given in the Result section. Adult female bioassay was divided into three sub-tests: (1) each plant species was tested against clean air, respectively, (2) a tobacco plant (as a reference) was tested against a plant from each of the other species. To ensure the non-bias of the setup, a tobacco plant was also tested against another tobacco plant, where one arbitrary side was considered as a reference, and (3) a tobacco plant (as a reference) was tested against two-plant complex (a tobacco plant together with a plant from each of the other plant species), to investigate possible masking effect of the volatile substances emitted from the other plant species on tobacco volatiles. Additionally, one tobacco plant was also tested against two tobacco plants complex. Larval choice response bioassay was also divided into three sub-tests: (1) a leaf disc prepared from each plant was tested against a similar-sized green filter paper disc, (2) a tobacco leaf disc *versus* a leaf disc of the other plant species or another tobacco leaf disc, and (3) a tobacco leaf disc was tested against another tobacco leaf disc plus a leaf disc of other plant species or two tobacco leaf discs. In the treatments involved in complex leaf discs, two leaf discs were placed next to each other.

### Statistical analysis

All statistical analyses were performed using SPSS 19.0 for Windows. Egg count data were square-root transformed, and then a paired *t* test was used to analyze within-group difference. Yate-corrected chi-square test was used to analyze within-group difference of larval choice frequencies. All tests were two-tailed, and the level of significance was set at α < 0.05.

## Results

### Oviposition choice response: Each plant species *versus* clean air

**Fig 2:**
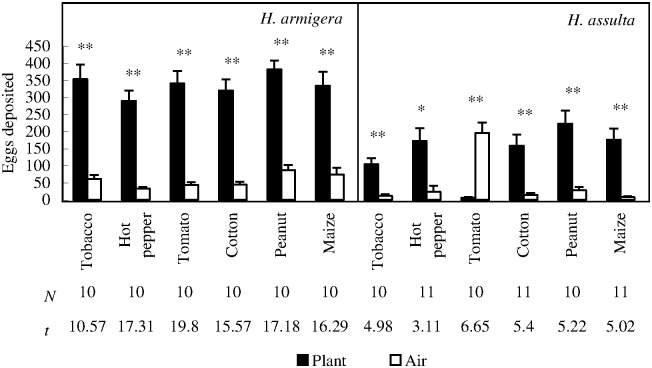
Ovipositional choice responses of *H*. *armigera* and *H*. *assulta* mated females under the conditions of each plant species paired with clean air. “*” and “**” at the upper panel of the figure indicate significant difference within group at *P* = 0.05 and *P* = 0.01 levels, respectively, tested by paired *t* test. The values of “*N*” and “*t*” beneath respective bars indicate replications and statistical *t* values, respectively.

*H*. *armigera* females laid significantly more eggs on all the plant odor sources than those on corresponding clean air controls (Fig 2; Paired *t* test, *P* < 0.0001 in all the cases). Percentage of eggs deposited on plant odor source ranged from 89.67% (tobacco) to 81.41% (peanut). Similarly, *H*. *assulta* females also laid significantly more eggs on their major host plants (tobacco and hot pepper) than those on corresponding clean air controls (Fig 2; Paired *t* test, *P*_tobacco_ = 0.0008, *P*_hot pepper_ = 0.0111). *H*. *assulta* eggs deposited on three non-host species (cotton, peanut, and maize), however, were also significantly more than those on corresponding clean air controls (Fig 2; Paired *t* test, *P* = 0.0005 in all the cases).

The most striking feature of *H*. *assulta* ovipositional bioassay is that, the females laid significantly less eggs on tomato odor source than those on corresponding clean air controls (Fig 2; Paired *t* test, *P* = 0.0001). Mean (±*SE*) eggs deposited on the former were only 5.67 ± 2.80, while those deposited on the latter were 195.67 ± 30.33.

### Oviposition choice response: Each plant species *versus* a tobacco plant

**Fig 3:**
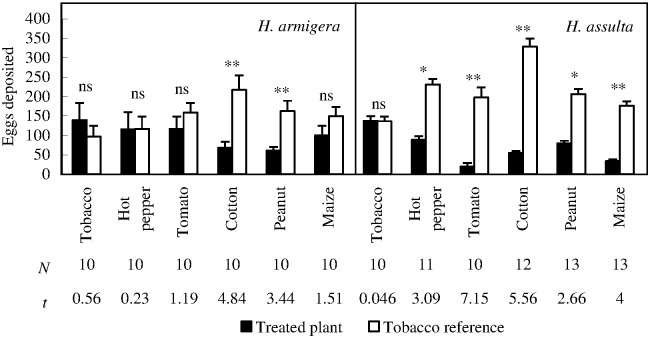
Ovipositional choice responses of *H*. *armigera* and *H*. *assulta* mated females under the conditions of each plant species paired with tobacco plant. “*” and “**” at the upper panel of the figure indicate significant difference within group at *P* = 0.05 and *P* = 0.01 levels, respectively, and “ns” indicates no significant difference within group, tested by paired *t* test. The values of “*N*” and “*t*” beneath respective bars indicate replications and statistical *t* values, respectively.

*H*. *armigera* females laid significantly less eggs on peanut and cotton than those on corresponding tobacco references (Fig 3; Paired *t* test, *P*_peanut *vs*. tobacco_ = 0.0073, *P*_cotton *vs*. tobacco_ = 0.0009), and no significant difference was found in the other pairs.

*H*. *assulta* females laid significantly more eggs on tobacco odor source than those on all the other plant odor sources (Fig 3; Paired *t* test, *P*_hot pepper *vs*. tobacco_ = 0.0115, *P*_peanut *vs*. tobacco_ = 0.0209, *P*_maize *vs*. tobacco_ = 0.0018, *P*_cotton *vs*. tobacco_ = 0.0002, *P*_tomato *vs*. tobacco_ = 0.0001). Percent eggs deposited on tomato odor source was as low as 8.87% when tomato was tested against tobacco.

When one tobacco plant was tested against another, the eggs deposited by both *H*. *armigera* (Fig 3; Paired *t* test, *P* = 0.5865) and *H*. *assulta* (Fig 3; Paired *t* test, *P* = 0.9646) showed no significant difference, suggesting no bias in our bioassay setup.

### Oviposition choice response: Two-plant complex *versus* a tobacco plant reference

**Fig 4:**
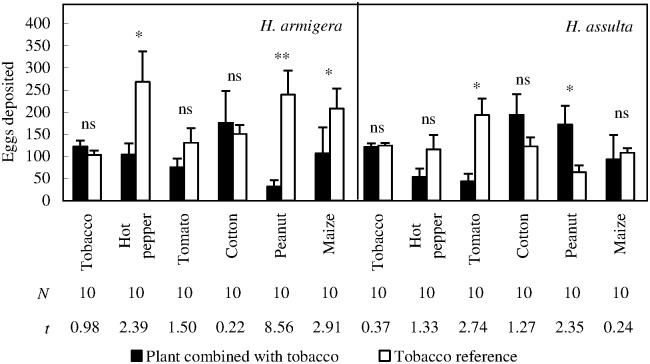
Ovipositional choice responses of *H*. *armigera* and *H*. *assulta* mated females under the conditions of each plant species combined with tobacco *versus* tobacco plant. “*” and “**” at the upper panel of the figure indicate significant difference within group at *P* = 0.05 and *P* = 0.01 levels, respectively, and “ns” indicates no significant difference within group, tested by paired *t* test. The values of “*N*” and “*t*” beneath respective bars indicate replications and statistical *t* values, respectively.

*H*. *armigera* females laid significantly less eggs on the odor source of peanut + tobacco complex than those on corresponding tobacco reference (Fig 4; Paired *t* test, *P* < 0.0001), and the eggs deposited on complex odor source only accounted for 11.84% of total eggs deposited. Similar results were obtained from the odor sources containing hot pepper and maize (Fig 4; Paired *t* test, *P*_hot pepper + tobacco *vs*. tobacco_ = 0.0407, *P*_maize + tobacco *vs*. tobacco_ = 0.0172). Tomato and cotton did not affect the egg distribution of *H*. *armigera* females.

*H*. *assulta* females laid significantly less eggs on tomato + tobacco complex odor source than on tobacco reference (Fig 4; Paired *t* test, *P* = 0.0134), and percent eggs deposited on complex odor source was as low as 18.39%; on the contrary, *H*. *assulta* females laid significantly more eggs on the odor source of peanut + tobacco complex than those on the tobacco reference (Fig 4; Paired *t* test, *P* = 0.0435). All the other plant species did not affect the egg distribution of *H*. *assulta* females.

Neither *H*. *armigera* nor *H*. *assulta* females deposited significantly more eggs on the odor source of two tobacco complex than those on the odor source of one tobacco plant (Fig 4; Paired *t* test, *P*_*H*. *armigera*_ = 0.3506, *P*_*H*. *assulta*_ = 0.7219).

### Larval choice response: A plant leaf disc *versus* green filter paper disc

**Fig 5:**
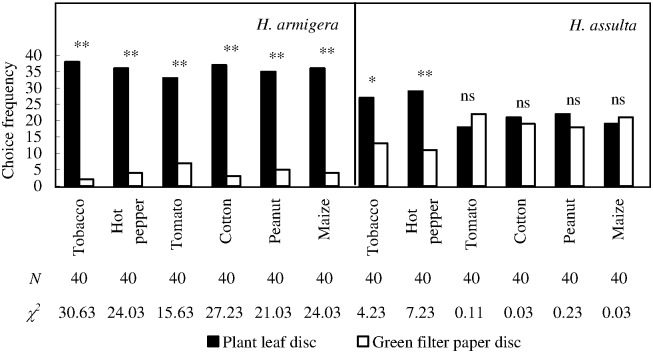
Choice responses of *H*. *armigera* and *H*. *assulta* larvae under the conditions of each plant leaf disc paired with green filter paper disc. “*” and “**” indicate significant difference within group at *P* = 0.05 and *P* = 0.01 levels, respectively, and “ns” indicates no significant difference within group, according to chi-square test. The values of “*N*” and “χ^2^” beneath respective bars indicate number of test insects and statistical χ^2^ values, respectively.

*H*. *armigera* larvae approached to all plant discs significantly more frequently than corresponding green filter paper discs (Fig 5; χ^2^ test with Yate-correction, *P*_tomato_ = 0.0001, *P* < 0.0001 in all the other cases). The percentage of ‘correct’ choice ranged from 95% (tobacco leaf disc) to 82.5% (tomato leaf disc).

*H*. *assulta* larvae also approached to tobacco and hot pepper leaf discs (major host plants) significantly more frequently than corresponding control discs (Fig 5; χ^2^ test with Yate-correction, *P*_tobacco_ = 0.0397, *P*_hot pepper_ = 0.0072), although the percentages of ‘correct’ choice were fairly low (tobacco: 67.5%; hot pepper: 72.5%). However, *H*. *assulta* larvae could not discriminate all the non-host leaf discs from corresponding control discs.

### Larval choice response: Each plant leaf disc *versus* a tobacco disc

**Fig 6:**
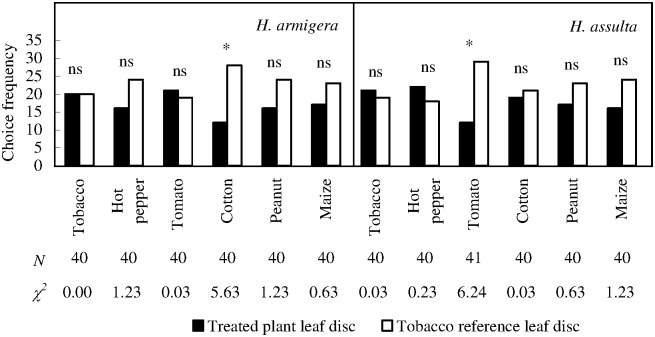
Choice responses of *H*. *armigera* and *H*. *assulta* larvae under the conditions of each plant leaf disc paired with tobacco leaf disc. “*” indicates significant difference within group at *P* = 0.05 level, and “ns” indicates no significant difference within group, according to chi-square test. The values of “*N*” and “χ^2^” beneath respective bars indicate number of test insects and statistical χ^2^ values, respectively.

*H*. *armigera* larvae approached to cotton leaf disc significantly less frequently than tobacco reference (Fig 6; χ^2^ test with Yate-correction, *P* = 0.0177), and they could not discriminate all the other leaf discs from corresponding references.

*H*. *assulta* larvae approached to tomato leaf disc significantly less frequently than tobacco reference (Fig 6; χ^2^ test with Yate correction, *P* = 0.0125). No other within-group difference was found.

### Larval choice response: Two-leaf disc complex *versus* a tobacco leaf disc

**Fig 7:**
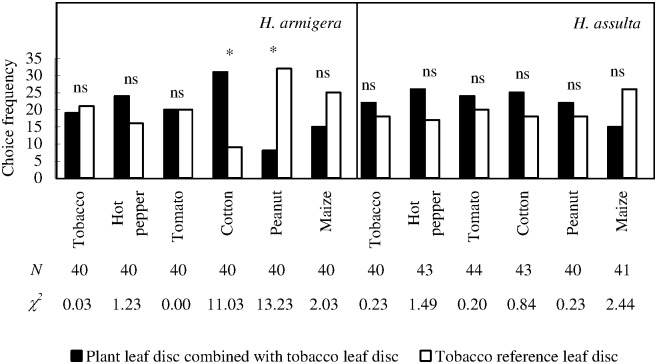
Choice responses of *H*. *armigera* and *H*. *assulta* newly hatched larvae under the conditions of each plant leaf disc combined with tobacco leaf disc *versus* tobacco leaf disc. “*” indicates significant difference within group at *P* = 0.05 level, and “ns” indicates no significant difference within group, according to chi-square test. The values of “*N*” and “χ^2^” beneath respective bars indicate number of test insects and statistical χ^2^ values, respectively.

*H*. *armigera* larvae approached to peanut + tobacco complex leaf discs significantly less frequently than tobacco reference (Fig 7; χ^2^ test with Yate-correction, *P* = 0.0003). The choice frequencies of the larvae to complex leaf discs and reference leaf disc were 8 and 32, respectively. On the contrary, cotton + tobacco complex leaf disc was chosen significantly more frequently than tobacco reference (Fig 7; χ^2^ test with Yate correction, *P* = 0.0009), and the choice frequencies of complex leaf disc and reference leaf disc were 31 and 9, respectively. No significant difference was found in within-group comparisons of *H*. *assulta* larvae.

### Comprehensive comparison

To make a comprehensive comparison between insect species, between stages, and among bioassay subsets, we summarized all the results obtained from Figs 2–7 in Table 1. Both stages of *H*. *armigera* and *H*. *assulta* could discriminate their corresponding host plants from corresponding controls (clean air or green filter paper disc). The other four plant species without unequivocal host status, however, elicited obviously different response patterns between the two stages of *H*. *assulta*: adult females mistakenly recognized all the plants but tomato as their hosts, while the larvae could not discriminate all the plant odor sources from corresponding controls.

**Table 1:** Comprehensive comparison: species, stages, and bioassays.

Test plants	Stronger than blank?		Differed from tobacco?		Masking tobacco volatile?	
	*H*. *armigera*: Adult / Larva	*H*. *assulta*: Adult / Larva	*H*. *armigera*: Adult / Larva	*H*. *assulta*: Adult / Larva	*H*. *armigera*: Adult / Larva	*H*. *assulta*: Adult / Larva
Tobacco	Y / Y	Y / Y	N / N	N / N	N / N	N / N
Hot pepper	Y / Y	Y / Y	N / N	L / N	Y / N	N / N
Tomato	Y / Y	D / N	N / N	L / L	N / N	Y / N
Cotton	Y / Y	Y / N	L / L	L / N	N / S	N / N
Peanut	Y / Y	Y / N	L / N	L / N	Y / Y	S / N
Maize	Y / Y	Y / N	N / N	L / N	Y / N	N / N

“Y” in the whole table means that the answer of the corresponding question in the first row was “Yes” judged by paired *t* test (adult) or chi-square test (larva), “N” means that the answer was “No” and the difference was not significant, “D” means significant deterring effect, “L” in the second data subset means response to the plant in the first column was significantly “lower” than to tobacco, and “S” in the third data subset means significant “synergic effect”.

When each plant species was tested against tobacco, the host selection patterns between the two stages of *H*. *armigera* were highly consistent (with the exception of peanut versus tobacco). In contrast, *H*. *assulta* females preferred tobacco to all the other plant species, while the larvae could not discriminate tobacco from all plant species but tomato.

Under complex plant odor sources, ovipositional choice response of *H*. *armigera* females to the complex odor sources involved hot pepper, peanut, and maize are decreased, compared with tobacco reference. Peanut, when placed together with tobacco, could reduce the approaching response of *H*. *armigera* larvae, compared with tobacco reference, while cotton played an opposite role (synergic effect). Tomato, which showed obvious ovipositional deterrence to *H*. *assulta* females in the first bioassay subset, again played a negative role when combined with tobacco. On the contrary, peanut, a plant species has never been recorded as a host of *H*. *assulta*, could enhance ovipositional response of the adult females when combined with tobacco. The host selection of *H*. *assulta* larvae was not disrupted by any plant species.

## Discussion

### Plant volatiles *versus* control

Our first hypothesis is fully supported by the two stages of two *Helicoverpa* species. However, *H*. *assulta* females could only recognize tomato as non-host plant, exhibiting a supersensitive response; instead, conspecific larvae could only detect their major hosts (tobacco and hot pepper) and could not actively avoid volatiles emitted from potential non-host plants, exhibiting an insensitive response (Fig 5, see also in S2 Dataset).

Different host selection response patterns between the two stages of *H*. *assulta* can be explained by at least three mechanisms: partial or incomplete host-related cues, risk-spreading ovipositional strategy [15], and the conservation or reappearance of olfactory receptors in *Helicoverpa* genus [16]. Firstly, in lepidopteron species, post-alighting cues and gustatory cues may be important in host selection of ovipositing females (for reviews, see [4, 17]) and feeding decision of larvae (e.g. *Manduca sexta* caterpillar, [18]), respectively. However, our bioassay protocols do not allow us to determine the relative importance of these cues. As a specialist, *H*. *assulta* newly hatched larvae are small and relatively immobile, so the ovipositing female often single-handedly makes the choice of larval food. Therefore, the larvae do not need to develop strong avoidance to non-host plants, this may contribute to the insensitive response of the larvae when host-related cues are partial or incomplete. Host acceptance or reject of ovipositing females is mainly depended on post-alighting cues in nature [4], thus they exhibited supersensitive response when only pre-alighting cues are available. Secondly, the ‘ovipositional mistakes’ of *H*. *assulta* females may be a risk-spreading strategy. That is to say, this behavior may be adaptive at population level if it allows to maintain a potential of host range expansion when host environment is undetectable or unpredictable. Thirdly, an electrophysiological study reported that several functionally similar olfactory receptors in three related species (*Heliothis virescens*, *H*. *armigera*, and *H*. *assulta*) are evolutionarily conserved, independent of the evolution of polyphagy and oligophagy [16]. This may be an alternative explanation to the supersensitive response exhibited by *H*. *assulta* ovipositing females in our study. Supersensitive host selection response of adult females in parallel with insensitive response of conspecific larvae has also been demonstrated in several specialist herbivores [19–21].

### Each plant species *versus* tobacco

Our second hypothesis is supported by the results obtained from ovipositing females of both species, since inter-specific comparison obviously indicates that the specialist exhibited more specialized ovipositional response to their shared host (tobacco) than its generalist sibling. However, this hypothesis is not supported by larval bioassay in most cases (Table 1).

Just like the first subset of bioassay, host selection response patterns of *H*. *armigera* females and larvae are highly consistent, with the exception of peanut. Neither the adult female nor the larvae of *H*. *armigera* preferred cotton materials, in accordance with previous study ([22], and References therein).

It seems that host-selection accuracy of *H*. *assulta* females in this situation was higher than that obtained from bioassay of plant *versus* clean air. This may probably be due to the hierarchy of host selection process of herbivorous insect: nevertheless, non-host plants were better than nothing. *H*. *assulta* larvae could only discriminate between tomato and tobacco, suggesting that host selection accuracy of the adult females was much stronger than that of conspecific larvae, in consistent with the reports of *Trichoplusia ni* [23] and *Melitaea athalia* [24]. However, this conclusion should better be made with caution, because of the different mobilities (crawling or flying) and bioassay methods between the two stages.

### Complex plant environment

The third hypothesis is partially supported by the adult females of both species, as well as by *H*. *armigera* larvae, but should be ruled out in the case of *H*. *assulta* bioassay. Peanut, hot pepper, and maize, which showed strong ovipositional attractiveness to *H*. *armigera* females when tested against clean air, could mask tobacco volatile in this situation, suggesting that two plant species, even both are ever-recorded hosts, also could play a masking role in host selection of a herbivorous insect via volatile exchange. This phenomenon is very common in nature but has received relatively little attention. Comparison between the second and the third subsets of bioassays indicates that, neither the relative ovipositional preference nor plant taxonomical relation have correlations with the presence or absence of this effect. The masking effect of tomato volatile on tobacco volatile with respect to host selection of *H*. *assulta* females could be explained by a simple mechanism: the oviposition deterrence of tomato outweighed the ovipositional attractiveness of tobacco plant. Since we excluded visual and tactile cues in adult oviposition bioassay, and the plants were planted in separate pots when they were needed to present together, the masking effect on tobacco could be explained by the alteration of volatile profiles from tobacco leaves by these plants.

Interestingly, peanut, a non-host plant of *H*. *assulta*, could help the adult females to locate to its ancestor host, tobacco, and plays an opposite role in tobacco-searching of the two sibling noctuid moths. Additionally, it is the only host plant which played a negative role in tobacco-approaching of *H*. *armigera* larvae. Volatile compounds emitted from undamaged and uninfected living peanut plant included (*E*)-4, 8-dimethyl-1, 3, 7-nonatriene, β-ocimene, linalool, and (*E*, *E*)-4, 8, 12-trimethyl-1, 3, 7, 11-tridecatetraene [25]. Among these, selective olfactory receptor neurons to (*E*)-β-ocimene and (*E*, *E*)-4, 8, 12-trimethyl-1, 3, 7, 11-tridecatetraene were shared by *H*. *assulta* and *H*. *armigera* [16, 26].

Our second subset of bioassay confirmed that cotton was not preferred by both stages of *H*. *armigera*. However, in the third subset of bioassay, cotton is the only host plant species that acted synergically on approaching of *H*. *armigera* larvae when combined with tobacco. A possibility is that, both tobacco volatile and cotton volatile were ‘suboptimal’ by themselves in terms of their attractiveness, but the mixed volatiles interacted complementarily. This is supported by a previous study conducted on the same insect species [27]. In that study, eight electrophysiologically active compounds were identified in the headspace of a primary host of *H*. *armigera*, pigeon pea, in relatively high concentrations. Other tested host plants, including tobacco and cotton used in our study, had a smaller subset of these compounds, all at relatively lower concentrations than pigeon pea. Headspace volatiles of various cotton varieties in their undamaged and uninfected status have been reported previously [28–31], which active compound(s) from cotton leaves acted complementarily with tobacco leaves need to be determined in the future.

### Tomato is not a host plant of *H*. *assulta*

The most striking result obtained in our study is that, tomato, a solanacous species, is the only one that could be recognized by *H*. *assulta* females as non-host plant (eggs deposited on tomato odor source: 5.67±2.80; on clean air source: 195.67±30.33, Fig 2 and Table 1, see also in S1 Dataset) and again the only plant species that could mask tobacco volatile among all the test plant species (Fig 4 and Table 1). Furthermore, it is the only plant species that elicited significantly lower response of *H*. *assulta* larvae than tobacco under the condition of tobacco *versus* each plant species (Fig 6 and Table 1). It has been demonstrated that neither detached tomato leaves nor potted plants did support growth of *H*. *assulta* neonates, and green-fruit-reared third instar larvae all died before sixth instar [14]. These authors attributed this larvicidal effect to a secondary metabolite, tomatine. In contrast, in this study, we found that the ovipositional deterrence of volatiles emitted from undamaged plant alone seems sufficiently to explain the non-host nature of tomato, because the larvae would have no chance to encounter tomatine in nature if their mothers do not lay eggs on tomato. β-Phellandrene is the dominant component in the headspace of different tomato genotypes [32–34]. We speculated that this compound may be responsible for the ovipositional deterrence of tomato to *H*. *assulta* females, but further chemical analysis and bioassay are needed. Additionally, the relationship between emission rate of tomato ovipositional deterring volatiles and *in vivo* tomatine content should be explored in the future. For example, *Depressaria pastinacella* larvae could avoid host-derived toxic furanocoumarins by virtue of their behavioral response to host-emitted octyl butyrate [35].

## Conclusion and implications

All together, we can make the following conclusions: (1) Under the conditions of plant materials *versus* neutral substrates, both stages of both *Helicoverpa* species could find their corresponding host plants. However, *H*. *assulta* females and larvae exhibited a supersensitive and an insensitive response, respectively, (2) in complex plant environment, two attractive host plants also can act antagonistically with respect to host selection of *H*. *armigera* via volatile exchange. Peanut played an opposite role in the two *Helicoverpa* species in their ovipositional responses to tobacco. Cotton can enhance the approaching response of *H*. *armigera* larvae when combined with tobacco, and (3) Tomato should be considered as a non-host plant of *H*. *assulta*.

These conclusions will help to deploy intercrops scientifically in agricultural practice. For example, suppression of *H*. *assulta* eggs deposited on tobacco via intercropping with tomato, or establishment of a push-pull system (for a review, see [36]) using tomato and peanut. The potential of hot pepper and maize in disrupting host location of *H*. *armigera* females in tobacco fields deserves further attention. Furthermore, since tobacco and cotton overlap phenologically in North-China, tobacco may become an effective refuge crop for mitigating *Bt* resistance of *H*. *armigera* in cotton fields. Several previous studies indicated that flowering cotton and tobacco are much more attractive to the adults of these *Helicoverpa* species than these crops in vegetative stages [7, 13, 37, 38]. However, our laboratory bioassays do not allow us to test these flowering plants without cutting them, further testing (taking into account pre- and post-alighting behaviors) in more realistic semi-field environment is underway.

## Supporting information

S1 Dataset Ovipositional choice response of adult females. (XLS)

S2 Dataset Larval approaching response. (XLS)
